# Increased Serum C3 and Decreased UA in Patients of Bipolar Disorder in Chinese Han Population

**DOI:** 10.3389/fpsyt.2018.00381

**Published:** 2018-08-21

**Authors:** Xiudeng Yang, Huai Tao, Ledong Xiao, Cunyan Li, Yamei Tang, Yong Liu

**Affiliations:** ^1^Department of Laboratory Medicine, The First Affiliated Hospital of Shaoyang University, Shaoyang, China; ^2^Department of Biochemistry and Molecular Biology, Hunan University of Chinese Medicine, Changsha, China; ^3^Department of Laboratory Medicine, Hunan Provincial People's Hospital, The First Affiliated Hospital of Hunan Normal University, Changsha, China; ^4^Department of Laboratory Medicine, The Second Xiangya Hospital, Central South University, Changsha, China; ^5^Department of Psychiatry, The Second Xiangya Hospital, Central South University, Changsha, China; ^6^Mental Health Institute of Central South University and Hunan Key Laboratory of Psychiatry and Mental Health, Changsha, China; ^7^China National Clinical Research Center on Mental Disorders (Xiangya) and China National Technology Institute on Mental Disorders, Changsha, China

**Keywords:** bipolar disorder, C3, C4, hypersensitive C-reactive protein, uric acid

## Abstract

The aim of this study is to explore the changes and clinical significance of serum C3, C4, hypersensitive C-reactive protein (hsCRP) and uric acid (UA) in patients of bipolar disorder (BD). In this case-control study, we recruited 141 BD patients from The Second Xiangya Hospital, Central South University, and 151 age and gender matched healthy controls (HC) from the health management central of The Second Xiangya Hospital. These patients were divided into two subgroups based on medicines use: 91 patients were treated with psychiatric drugs and 50 patients were drugs free, or four subgroups based on mood states: 54 patients in manic/hypomanic phase, 30 patients in depressive phase, 52 patients in euthymic phase and 5 patients in mixed phase. Serum levels of C3, C4, hsCRP and UA were measured in all subjects. The serum C3 levels in BD patients (0.9981 ± 0.1849 g/L) were significantly lower than that in HC group (1.0637 ± 0.2186 g/L), especially the drugs free subgroup and the euthymic subgroup (0.975 ± 0.153 and 0.983 ± 0.182 g/L), while the serum UA levels were significantly higher (354.6 ± 90.4 vs. 332.9 ± 88.7 μmol/L), especially the drug-treated subgroup and manic/hypomanic subgroup (361.56 ± 93.20 and 376.70 ± 88.89 μmol/L), and rates of hyperuricaemia (31.91 vs. 17.88%) were significantly higher in BD patients than in HC group. The serum C4 and hsCRP levels in HC group showed no significant difference with BD patients in whole or those subgroups. These findings suggested that the complement and purinergic systems of BD patients might be disrupted, the UA levels could be a potential marker in manic phase and the C3 might be the marker of therapeutic evaluation of BD patients.

## Introduction

Bipolar disorder (BD) is one of the severe mental disorders with a lifetime prevalence of 2.4% featured with the recurrence of depressive/manic episodes as well as mixed states (1). However, the etiology of BD remains uncertain. The immune-inflammatory has been suggested to play a role in the etiology of BD (2). In the previous investigation, the levels of inflammatory-associated cytokines were found increased both in the brain and peripheral serum in BD patients (3). C-reactive protein (CRP) is a pentameric acute-phase protein that produced in the liver and secreted into the blood. CRP plays a prominent role in the innate immune system, as it increase rapidly in response to infection and inflammatory stimulation, then reduced sharply after the acute phase (4). Fernandes et al. (5) found that serum/plasma CRP levels are increased in BD patients than health control (HC) group regardless of mood states, particularly the CRP levels of those patients in manic/hypomanic phase were much higher than in depressive and euthymic phases. The complement system is one of the most important components of humoral immunity participating in both adaptive and acquired immune responses. CRP activates the classical complement pathway, and then stimulates phagocytosis via binding to Fc immunoglobulin receptors as an opsonin (4). Even though their importance in immune functions, complement factors have been barely investigated and levels of these factors among different stages of BD have not been compared. As inflammation responses often involve the complement system activation, we hypothesized that complement system factors might be related with the symptom severity of BD. Akcan et al.(6) found serum complement (C4, factor B, and sC5b-9) levels were significantly reduced in chronic BD patients when compared with HC group, while the peripheral blood mononuclear cell mRNA expression levels of C1q and C4 were significantly elevated. Thus, the detection of different components involved in this disorder might provide new possibilities of treatment, as it was reported that the adjunctive addition of anti-inflammatory medications with lithium has achieved complete remission in treating BD (7).

Recently, increasing evidences showed that BD might be closely associated with the dysfunction of adenosine and purinergic systems (8, 9). Adenosine is one of the purine nucleosides and appears to modulate some neurotransmitters, which has attracted our attention into understanding the pathophysiology of this disease that deep-rooted in human central nervous system (10). Uric acid (UA) is the product of xanthine or hypoxanthine catalyzed by xanthine oxidoreductase and the end product of purine nucleosides metabolism. The central UA levels have a strong positive association with peripheral levels (11), and the increased serum UA levels may be the sign of over-activation of purinergic system. A meta-analysis studied by Bartoli et al. (12) found that subjects with BD have higher UA levels than that in HC, especially in the manic/hypomanic and mixed phases. Recently, in a randomized, placebo-controlled study, a xanthine oxidase inhibitor allopurinol that used for gout and hyperuricemia was effective both in reducing serum UA levels and improving manic symptoms of BD patients (13).

The trait marker and state marker of each phase of BD has not been established. Therefore, the purpose of current study was to measure peripheral C3, C4, hsCRP, and UA levels in BD patients across the different mood states and look for whether levels of these indicators are correlated with the severity of mood symptoms. Besides, in order to find out if mood stabilizers could induce the changes of these parameters, we made a comparison between psychiatric drugs treatment and drugs free subgroup as well. In addition, considering this research has not been widely reported in Chinese Han population, we expect these results could provide potential diagnosis and treatment for BD patients in China.

## Materials and methods

### Subjects

One hundred and forty one BD patients were recruited from Department of psychiatry of the Second Xiangya Hospital, Central South University. Those patients were diagnosed by two senior psychiatrists according to criteria of the Diagnostic and Statistical Manual of Mental Disorders, Fifth Edition (DSM-V). As we presented in the Table 1, 91 of 141 BD patients were taking psychiatric medication: 85(93.4%) were taking mood stabilizers, 68(74.7%) were taking antipsychotic medications and 12(13.2%) were taking antidepressants. The exclusion criteria for patients were any comorbid mental disorders, a history of traumatic brain injury, intellectual disability, serious somatic diseases, active or chronic inflammatory, autoimmune diseases, pregnancy or breast feeding. 151 age and gender matched HC were recruited from the health management center of The Second Xiangya Hospital at the same period after negatively screening for the presence of a past or current psychiatric symptoms. The severities of mood symptoms were assessed using the 17-item Hamilton Depression Rating Scale (HDRS) and the 11-item Bech-Rafaelsdn Mania Rating Scale (BRMS). Sociodemographic features of the group are demonstrated in Table 1.

**Table 1:** Demographic data for BD patients and HC group.

	**BD patients**	**HC**	**t/χ^2^**	***P*-value**
Sex	N(%)	N(%)		
Male	61 (43.26)	67 (44.37)	0.036	0.906
Female	80 (56.74)	84 (55.63)		
Total	141	151		
Age(Mean ± SD)	29.11 ± 12.08	30.60 ± 11.04	1.101	0.272
**MOOD STATES**				
Mania/hypomania	54 (38.30)	/		
Depression	30 (21.27)	/		
Euthymia	52 (36.88)	/		
Mixed	5 (3.55)	/		
Drugs use(yes/no)	91/50	/		
HDRS(Mean ± SD)	18.04 ± 13.84	/		
BRMS(Mean ± SD)	22.80 ± 14.39	/		
Hyperuricemia	45 (31.91)	27 (17.88)	7.730	0.004

This study was approved by The Ethics Committee of The Second Xiangya Hospital, Central South University. All the patients or their statutory guardians and HC were required to sign an informed consent forms.

### Sample collection and processing

5 ml peripheral venous blood samples were drawn from fasting BD patients and HC in the morning and pro-coagulant tubes. Any sample with hemolysis was discarded and avoided repeatedly freezing-thawing. Peripheral serum were isolated after centrifuging at 3500 rpm for 10 min and stored at −80°C until analysis. All samples were measured after thawing to room temperature. The serum C3 and C4 levels were measured with immuno-scatter turbidmetric assay, in which C3 and C4 are combined with antibody to formed the complexes, hsCRP were measured with latex enhanced immunoturbidimetric assay in an automatic analyzer Abbott c8000, in which hsCRP are combined with monoclonal antibody attached on latex particle, and UA were analyzed by direct enzymatic method, in which UA was oxidized by uricase coupled with peroxidase in an automatic analyzer Hatichi 7600.

### Statistical analysis

Statistical analyses were performed with the SPSS (version 20.0). Data were presented as mean ± standard deviation (SD) for normal distribution variables (at Kolmogorov-Smirnov test) and median (quartile range) for non-normal distribution variables. Continuous variables were compared using Student's *t*-test or the Mann-Whitney U-test as appropriate. The rates of hyperuricemia (UA ≥ 420 μmol/L in male, UA ≥ 360 μmol/L in female) and gender distribution in these two study groups were compared with χ^2^ test. When baseline characteristics among three groups were compared, normally distributed continuous variables were compared with the one-way ANOVA and skewed distributed with Kruskal-Wallis H test. Comparison between two groups were considered statistically significant if *P* < 0.05, Bonferroni correction was used to adjust our results for multiple comparisons.

## Results

### Serum C3, C4, hsCRP, and UA levels in HC group and BD patients

**Figure 1:**
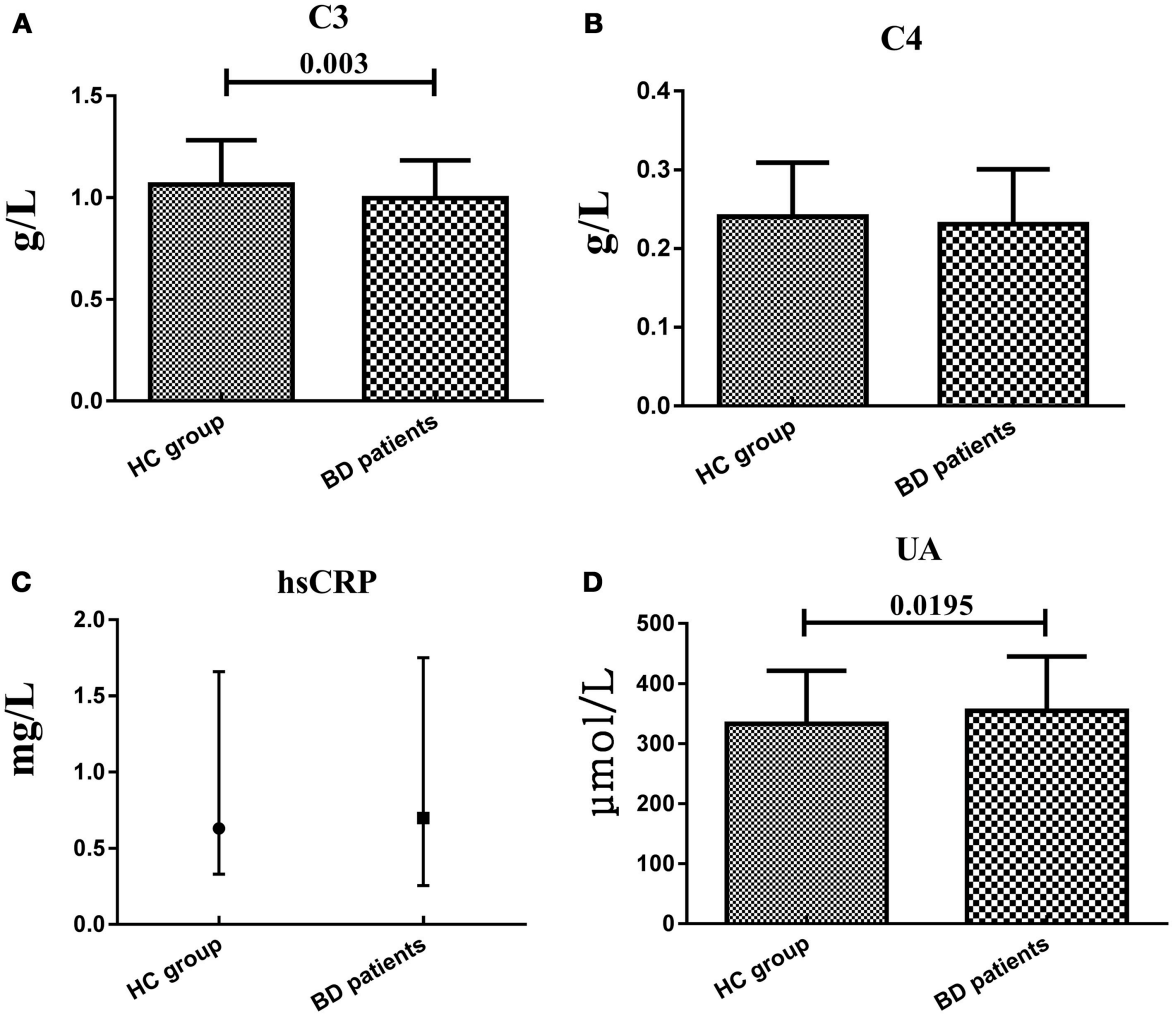
Representative of the serum C3, C4, hsCRP, and UA levels in two groups. **(A,B and D)** were presented in mean ± SD and **(C)** was presented in median value and interquartile range.

The mean age of the patients was 29.11 ± 12.08 years old, and 43.26% of the patients were males, and the HC was 30.60 ± 11.04 years and 44.37% were males. There were no significant difference in terms of age and gender between two study groups (*t* = 1.101, *df* = 290, *P* = 0.272; χ^2^ = 0.036, *df* = 1, *P* = 0.906). We enrolled 54 patients in manic/hypomanic state, 30 patients in depressive state, 52 patients in stable euthymia and 5 patients in mixed state. The serum C3, C4, hsCRP, and UA in BD patients and HC group were measured at the same time. Serum C3 levels in BD patients (0.998 ± 0.185 g/L) were significantly lower (*t* = 2.76, *df* = 290, *P* = 0.003) than that in HC group (1.064 ± 0.219 g/L), while the serum UA levels were significantly elevated (*t* = −2.068, *df* = 290, *P* = 0.0195) in BD patients (354.64 ± 90.37 vs. 332.96 ± 88.70 μmol/L). Meanwhile, the serum C4 and hsCRP levels showed no difference between two groups (0.240 ± 0.069 *vs*. 0.231 ± 0.070 g/L; 0.63 (0.33, 1.65) vs. 0.70 (0.26, 1.73) mg/L, respectively) as a whole (Figure 1). Both HDRS and YMRS scores showed no significant positive or negative correlation with serum C3, C4, hsCRP, and UA levels in BD patients.

### Serum C3, C4, hsCRP, and UA levels in drugs use/free group

**Figure 2:**
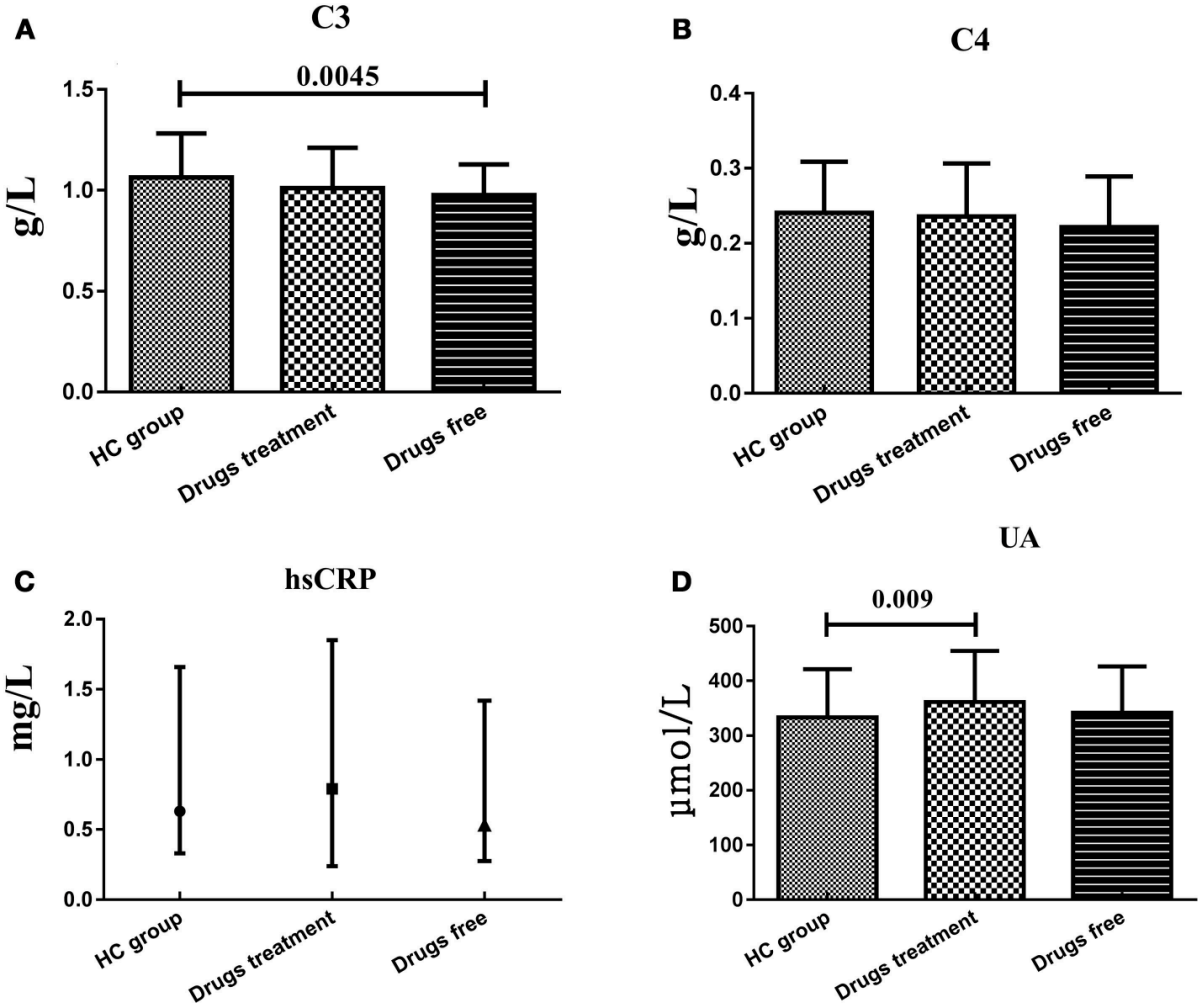
Representative of the serum C3, C4, hsCRP, and UA levels in HC group and BD patients in drugs use and drugs free subgroups. **(A)** Statistical results showing the comparison of serum C3 levels between HC group and drugs treatment/free subgroup. **(B)** Statistical results showing the comparison of serum C4 levels between HC group and drugs treatment/free subgroup. **(C)** Statistical results showing the comparison of serum hsCRP levels between HC group and drugs treatment/free subgroup. **(D)** Statistical results showing the comparison of serum UA levels between HC group and drugs treatment/free subgroup.

Patients were evaluated in two subgroups in terms of previous treatment: never used any treatment or stopped treatment for at least 1 month before participating in this study, and continued their treatment for at least 1 month. Serum C3 levels in the drugs free subgroup (0.975 ± 0.153 g/L) were significantly lower (*t* = 2.65, *df* = 199, *P* = 0.0045) than that in HC group (1.064 ± 0.219 g/L), while the drugs treatment subgroup (1.011 ± 0.200 g/L) showed no significant difference with HC group. Serum UA levels in drugs treatment subgroup (361.56 ± 93.20 μmol/L) were significantly higher (*t* = −2.384, *df* = 240, *P* = 0.009) than that in HC group (332.96 ± 88.70 μmol/L), while the drugs free subgroup (342.04 ± 84.44 μmol/L) showed no significant difference with HC group. However, serum C4 and hsCRP levels in both drugs treatment and free subgroups showed no significant difference with HC group (Figure 2).

### Serum C3, C4, hsCRP, and UA levels in different mood states

**Figure 3:**
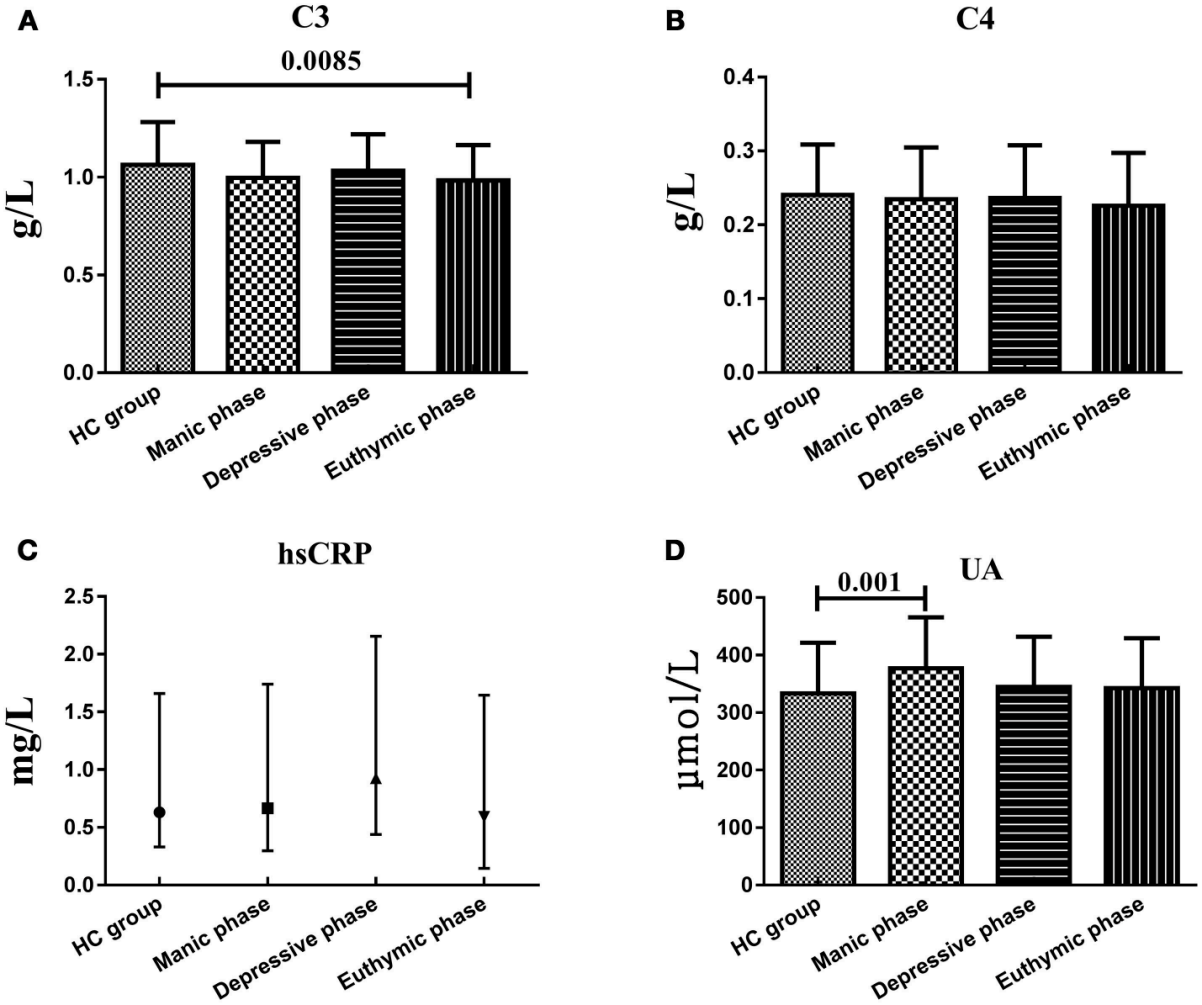
Representative of the serum C3, C4, hsCRP, and UA levels in HC group and BD patients in different mood states. **(A)** Statistical results showing the comparison of serum C3 levels between HC group and patients in different mood states. **(B)** Statistical results showing the comparison of serum C4 levels between HC group and patients in different mood states. **(C)** Statistical results showing the comparison of serum hsCRP levels between HC group and patients in different mood states. **(D)** Statistical results showing the comparison of serum UA levels between HC group and patients in different mood states.

Considering the small sample size, the patients in mixed state were not analyzed and compared with another three subgroups. There were no statistically significant difference of serum C3, C4, hsCRP, and UA levels between BD patients during different phases of illness, whereas instead of in another two subgroups, UA levels in manic/hypomanic phase (376.70 ± 88.89 μmol/L) were significantly higher (*t* = 3.109, *df* = 203, *P* = 0.001) than those of the HC (332.96 ± 88.70 μmol/L) and C3 levels in euthymic phase (0.983 ± 0.182 g/L) were significantly lower (*t* = −2.406, *df* = 201, *P* = 0.0085) than HC group (1.064 ± 0.219 g/L). Serum C4 and hsCRP levels in all these subgroups were not different with HC group (Figure 3).

### Serum C3, C4, hsCRP, and UA levels in different genders

**Figure 4:**
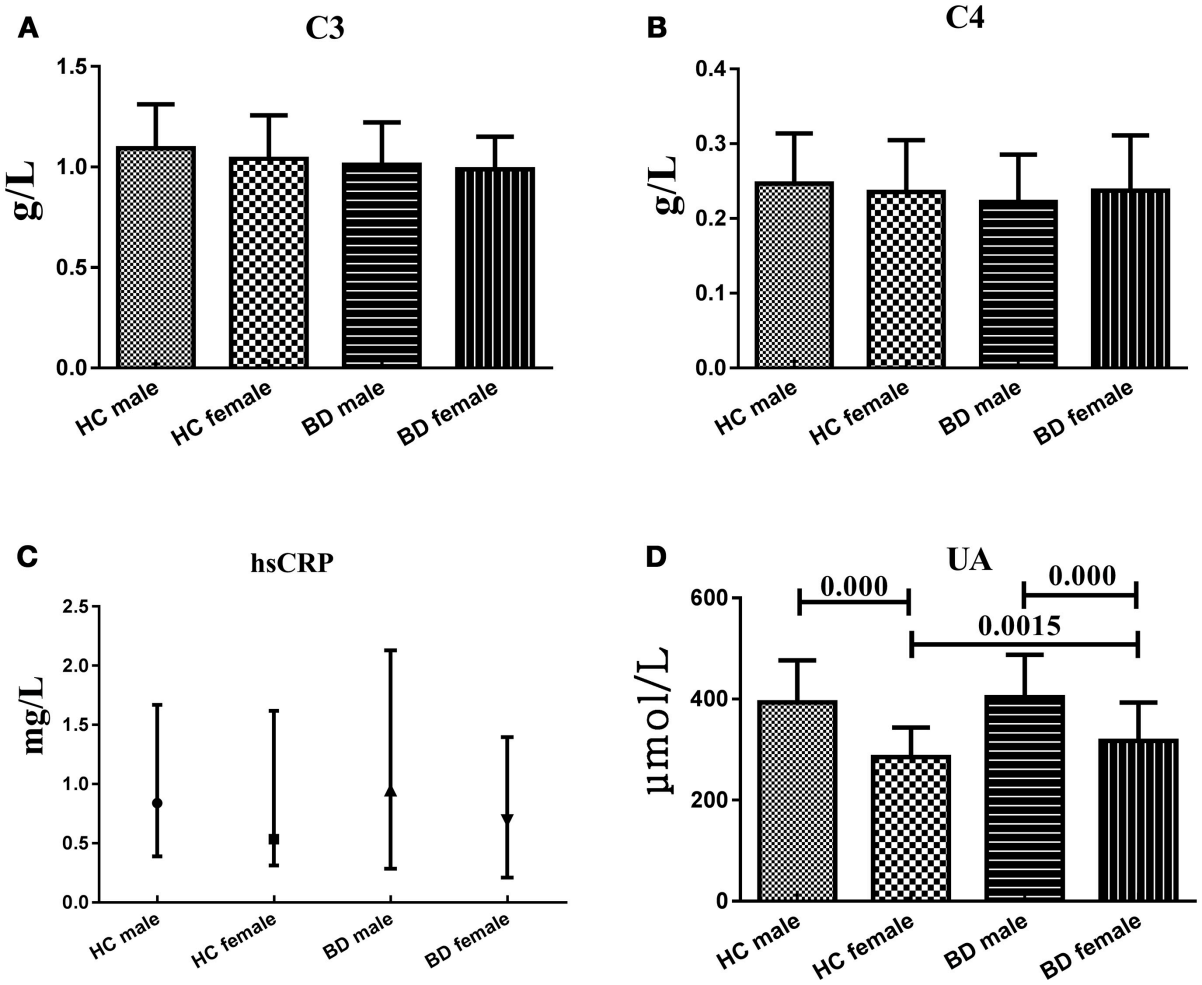
Representative of the serum C3, C4, hsCRP, and UA levels in HC group and BD patients in different genders. **(A)** Statistical results showing the comparison of serum C3 levels between HC group and BD patients in different genders. **(B)** Statistical results showing the comparison of serum C4 levels between HC group and BD patients in different genders. **(C)** Statistical results showing the comparison of serum hsCRP levels between HC group and BD patients in different genders. **(D)** Statistical results showing the comparison of serum UA levels between HC group and BD patients in different genders.

To explain the effect of different genders on the levels of serum C3, C4, hsCRP, and UA levels, we compared these indicators between two genders within HC group or BD patients and between two groups from the same gender. Serum C3, C4 and hsCRP, levels showed no significant difference between males and females both in HC group and BD patients, nor the difference between two groups from the same gender. However, serum UA levels in female HC (285.02 ± 58.48 μmol/L) were significantly lower (*t* = 9.328, *df* = 149, *P* = 0.000; *t* = 3.026, *df* = 162, *P* = 0.0015, respectively) than that in male HC (393.06 ± 83.59 μmol/L) and female BD patients (317.02 ± 76.21 μmol/L), and the serum UA levels in male BD patients (403.97 ± 83.96 μmol/L) were significantly higher (*t* = −6.423, *df* = 139, *P* = 0.000) than that in female BD patients (317.02 ± 76.21 μmol/L) (Figure 4).

## Discussion

In psychiatric diseases, the mechanism of complement system has most widely been studied in schizophrenia and other mental disorders with plenty of contradictory results published previously, while the role of complement components in BD were scarcely reported. C3 is the most abundant complement in serum and the pivotal of connecting the classical and alternative complement pathways, and its serum levels was followed by serum C4 levels. So, the fluctuation of serum C3 levels is paralleled with total content of complement factors and the measurement of serum C3 levels could partly reflect the change of the whole complement metabolisms. C4 is activated by C1s and then hydrolyzed to C4a and C4b in the alternative pathways. In this primary study, we found that serum C3 levels in BD patients were significantly lower than HC, while C4 were not changed obviously. Therefore, we hypothesized that the complement system failed to maintain dynamic balance basically in the BD patients, but not knowing which one of the three pathways were disturbed, since there was no change in C4 levels. The significantly reduced serum C3 levels in drugs free subgroup of BD patients, but not in drugs treatment subgroup, illustrated that psychiatric drugs were able to increase serum C3 levels, and we can postulate that the decreasing C3 levels might be the cause of BD rather than the results. However, in a study reported by Akcan et al.(6) found that serum C4 levels were significantly reduced in chronic BD patients and the C4 mRNA expressions were elevated in a compensatory way. Furthermore, Santos and his colleagues failed to detect significant difference between HC and BD patients in euthymic state(14). In spite of these conflict results, this would be understandable when considering the huge discrepancy of sample size and the subjects recruited. In addition, there was no significant difference of serum C3 levels among these subgroups in different mood states, so in general, this indicator were not recommended as the state biomarker of BD patients.

Over the past decade, some authors have described the possible participation of purinergic system dysfunction in the pathophysiology of BD (15, 16). More recently, the increased serum UA levels in BD patients of manic state have been widely reported (8, 17). In accordance with these available data, our results confirmed that BD patients showed significantly higher serum UA levels and higher rates of hyperuricemia than HC group. In a large and multi-centric, nationwide population-based epidemiological investigation that spanning six years, Chung found that the BD patients had the increased risk of hyperuricemia that resulting in gout, indicating that the similar neurobiochemical mechanism were shared between these two diseases (18). Therefore, UA may be a promising biomarker in BD. Besides, the serum UA levels of BD patients in drugs treatment subgroup and in manic/hypomanic state were higher than HC group, but not the drugs free subgroup and patients in depressive and euthymic state. So, we agree that the routine measurement of serum UA levels might be helpful to identify patients in manic/hypomanic state who may benefit from adjunctive treatment with purinergic modulators (16). However, Albert reported that serum UA levels were higher in BD patients never exposed to mood stabilizers than HC group (8), which was contrary to our results. We speculated that the duration of drugs treatment maybe one of the critical factors should be considered seriously, and the short-term treatment might not able to reduce serum UA levels obviously. We were in a cautious attitude toward whether the increase of serum UA levels resulted from purinergic dysfunctions was the trait maker of BD, as we failed to detect significant differences among these BD patients in manic/hypomanic, depressive and stable euthymic states. In sum, serum UA level may represent key target in the search for clinically relevant biomarkers.

In the previous studies, pro-inflammatory and inflammatory cytokines were found elevated in manic episode of BD (19, 20), indicating inflammatory cytokines contribute to the pathophysiology of BD. According to our results, the serum hsCRP levels in BD patients showed no significant difference with HC group, and the differences among three mood states were moderate. It was reported that serum hsCRP levels were significantly higher in manic BD patients before treatment than HC group, and were significantly decreased after treatment. Therefore, serum hsCRP levels maybe a potential indicator in predicting treatment outcomes (20). However, the serum hsCRP levels in drugs treatment subgroup were not different from drugs free subgroup. A possible explanation is that the baseline hsCRP levels among individuals differs obviously from each other while this cross-sectional research didn't compare the serum hsCRP levels before and after treatment. A recent study about the structural volume change of a specific brain region along with cognitive function demonstrated that the orbitofrontal cortex had a significantly negative correlation with serum hsCRP levels after adjustment for age and gender. And authors speculated that persistent inflammation indicated by elevated serum hsCRP levels in euthymic phase may involve the pathogenesis or pathophysiology of alteration of the frontal pathway (21). Although not statistically significant, the results of the studies underlined above emphasize the role of hsCRP on BD.

In sum, this original research involved more subjects than most of previous studies and patients were grouped based on whether they were treated or not with psychiatric drugs and which mood states they are in. As for the gender factors, we failed to find any differences of serum C3, C4, and hsCRP levels between two genders within the HC group or BD patients, or between the two groups within the same gender. However, the serum UA levels in male HC group and male BD patients were both significantly higher than female HC group and female BD patients, respectively. The significant gender difference within the same group could be explained by different lifestyle between males and females, like the diet, alcohol abuse, and cigarette smoking. The significantly higher serum UA levels in female BD patients than female HC group may attribute to the effect of estrogen on the UA metabolism.

Generally, some limitations and open questions requiring further research. The sample size of BD patients in mixed state was moderate, further research should involve more patients. In addition, we only measured the serum levels of those indicators, instead of the expression level. Even if we grouped these patient into drugs treatment subgroup and drugs free subgroup, the types of drugs, duration of administration and disease duration differed among each other. From this study, we could cautiously infer that UA could represent both a trait and state marker of BD, but whether a prognostic biomarker deserves further proof.

## Author contributions

YL designed the study. HT and LX acquired the data, which YT and CL analyzed. XY, HT, and LX read and wrote the manuscript. All authors reviewed and approved for publication.

### Conflict of interest statement

The authors declare that the research was conducted in the absence of any commercial or financial relationships that could be construed as a potential conflict of interest.
